# Application of Micro Quartz Tuning Fork in Trace Gas Sensing by Use of Quartz-Enhanced Photoacoustic Spectroscopy

**DOI:** 10.3390/s19235240

**Published:** 2019-11-28

**Authors:** Haoyang Lin, Zhao Huang, Ruifeng Kan, Huadan Zheng, Yihua Liu, Bin Liu, Linpeng Dong, Wenguo Zhu, Jieyuan Tang, Jianhui Yu, Zhe Chen, Frank K. Tittel

**Affiliations:** 1 Key Laboratory of Optoelectronic Information and Sensing Technologies of Guangdong Higher Education Institutes, Department of Optoelectronic Engineering, Jinan University, Guangzhou 510632, China; linhaoyang12@gmail.com (H.L.); hhhuangzhao@foxmail.com (Z.H.); liuyihua9701@foxmail.com (Y.L.); lpdong@jnu.edu.cn (L.D.); zhuwg88@163.com (W.Z.); tangjiey@163.com (J.T.); kensomyu@gmail.com (J.Y.); thzhechen@jnu.edu.cn (Z.C.); 2 State Key Laboratory of Applied Optics, Changchun Institute of Optics, Fine Mechanics and Physics, Chinese Academy of Sciences, Changchun 130033, China; rfkan@ciomp.ac.cn; 3 Guangdong Provincial Key Laboratory of Optical Fiber Sensing and Communications, Jinan University, Guangzhou 510632, China; 4 School of Physics and Optoelectronic Engineering, Foshan University, Foshan 528000, China; binliu@fosu.edu.cn; 5 Department of Electrical and Computer Engineering, Rice University, Houston, TX 77005, USA; fkt@rice.edu

**Keywords:** quartz tuning fork, photoacoustic spectroscopy, quartz-enhanced photoacoustic spectroscopy, acoustic detection module

## Abstract

A novel quartz-enhanced photoacoustic spectroscopy (QEPAS) sensor based on a micro quartz tuning fork (QTF) is reported. As a photoacoustic transducer, a novel micro QTF was 3.7 times smaller than the usually used standard QTF, resulting in a gas sampling volume of ~0.1 mm^3^. As a proof of concept, water vapor in the air was detected by using 1.39 μm distributed feedback (DFB) laser. A detailed analysis of the performance of a QEPAS sensor based on the micro QTF was performed by detecting atmosphere H_2_O. The laser focus position and the laser modulation depth were optimized to improve the QEPAS excitation efficiency. A pair of acoustic micro resonators (AmRs) was assembled with the micro QTF in an on-beam configuration to enhance the photoacoustic signal. The AmRs geometry was optimized to amplify the acoustic resonance. With a 1 s integration time, a normalized noise equivalent absorption coefficient (NNEA) of 1.97 × 10^−8^ W·cm^−1^·Hz^−1/2^ was achieved when detecting H_2_O at less than 1 atm.

## 1. Introduction

Trace gas sensing technology has been widely used in many fields including industrial processes control, medical diagnosis, environmental monitoring, detection of toxic gases, and breath analysis [1,2,3,4]. Non-optical techniques based on chemical sensing, gas chromatography/mass spectrometry., and electrochemistry have a high cost, a bulky structure, and a slow reaction rate. Most of the optical sensing techniques are cost-effective, compact, and able to operate in real time [5,6,7].

Photoacoustic spectroscopy (PAS) is a practical approach for optical trace gas sensing. The principle of PAS is based on the detection of the acoustic waves, which are generated by the variation of localized pressure and temperature upon the absorption of modulated optical radiation by the targeted medium [8,9]. An acoustic transducer such as a microphone, a cantilever, or a quartz tuning fork is employed to convert the acoustic signal into an electric signal. PAS signals can also be collected by a thin metal foil [10] or film [11]. The advantages of PAS are compact size, simplicity of use, and wide dynamic range. The PAS characteristics is that the acoustic transducer is not limited by the optical wavelength by the excitation sources, which leads to the fact that a commonly used microphone can be applied to from ultraviolet to infrared light sources [12]. This fact makes the PAS applications more wide-ranging and reduces the cost of PAS based instruments. PAS has been successfully applied to applications including atmospheric pollution monitoring, agricultural and industrial processes control, as well as medical diagnostics [13]. For example, PAS has been used to monitor nitric oxide (NO) in vehicle exhaust emissions [14] and methane (CH_4_) in atmospheric pollution [15].

As a variant of PAS, quartz-enhanced photoacoustic spectroscopy (QEPAS) has been used in numerous applications. QEPAS has rapidly developed since the invention by A. Kosterev in 2002 [16]. The principle of QEPAS is to use a sharply resonant quartz tuning fork (QTF) as an acoustic transducer to accumulate acoustic energy by means of piezoelectric effect. The amplitude of QEPAS signal is proportional to the gas concentration, the absorption coefficient, and the laser power [17]. The use of QTF, instead of conventional photoacoustic cells, reduced the limitations of acoustic resonance conditions [18]. A commercially available QTF has a resonant frequency of 32.7 kHz and a high Q factor of ~10^4^ at atmospheric pressure (~10^5^ Pa). The prominent features of QEPAS are an ultra-compact setup, low cost, and high noise immunity [19,20,21,22,23,24,25]. The high noise immunity of QEPAS can be attributed to the anti-symmetric vibration, a high-quality factor (Q factor), and the narrow resonant bandwidth of a QTF [26]. To further enhance the QEPAS signal, an acoustic micro-resonator (AmR) can be added to the QTF in on-beam [27] or off-beam configurations [28]. AmRs made of thin stainless tubes are acoustically coupled with the QTF to confine the acoustic waves and enhance the QEPAS performance. A part-per-trillion level detection of SF_6_ can be achieved by the use of QEPAS sensor [29].

Until 2013, custom-made tuning forks instead of commercially available QTFs were demonstrated for QEPAS detection by P. Patimisco et al. [30]. The large prong spacing and low resonance frequency benefit the use of mid-infrared lasers and the detection of molecules with low vibration-translation (V-T) rates [31]. Y. Ma demonstrated the use of 30.72 kHz QTF in spatially resolved gas detection [32]. J. Li demonstrated the use of 75 kHz QTF in the detection of broadband absorbers [33]. M. Duquesnoy demonstrated a custom QTF with a reduced fundamental frequency of 21.2 kHz and a large prong spacing of 2 mm [34]. Recently, a custom QTF with prong spacing up to 1.5 mm and a Q value of ~15,000 were employed for ppb-level ethylene detection by V. Spagnolo [35]. In our previous publication, a custom tuning fork with an optimal AmR enhances the detection signal-to-noise (SNR) by >100 times in comparison with a bare custom QTF [36].

In this manuscript, to the best of our knowledge, the most compact QEPAS sensor based on a micro QTF is reported. A micro QTF with a resonance frequency of 32.7 kHz and a dimension of 0.72 × 3.5 mm, which is 3.7 times smaller than that the usually used standard QTF, was employed as the photoacoustic transducer for trace gas analysis. The laser focus position effects and modulation depth were investigated to improve the QEPAS excitation efficiency. A pair of micro resonators was coupled with the micro QTF in an on-beam configuration to enhance the acoustic signal. The sensor configuration and resonator parameters were optimized to obtain maximum signal amplitude.

## 2. Characterization of the Micro Quartz Tuning Fork

**Figure 1:**
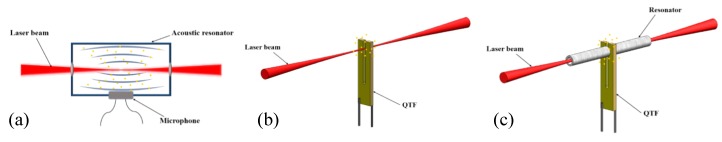
Schematic of (**a**) PAS spectrophone and QEPAS spectrophones based on (**b**) quartz tuning fork (QTF) and (**c**) on-beam configuration. PAS: Photoacoustic spectroscopy; QEPAS: Quartz-enhanced photoacoustic spectroscopy.

**Figure 2:**
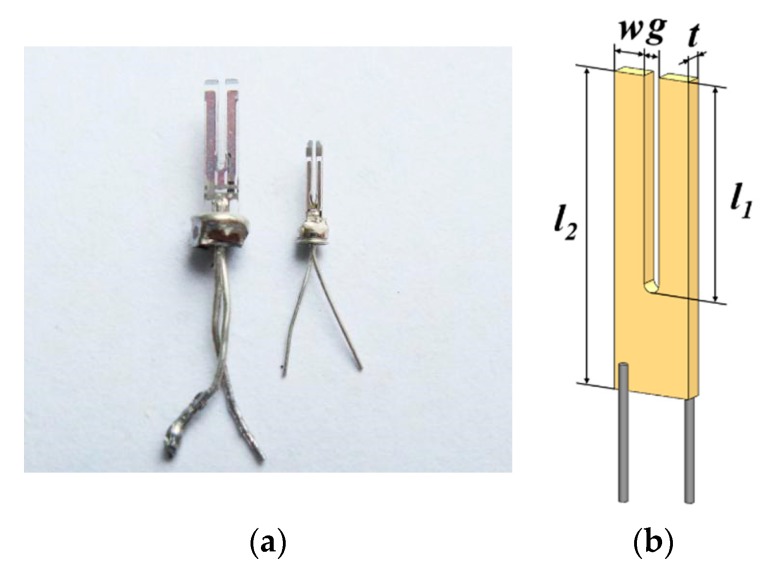
(**a**) Picture of micro QTFs and standard QTFs; (**b**) geometry of a QTF.

Conventional PAS spectrophone and QEPAS spectrophone based on bare QTF and on-beam configuration are shown in Figure 1a–c, respectively. The volume of a PAS resonator equipped with a microphone, shown in Figure 1a, was usually dozens of milliliters [9]. The using of QTF reduced the spectrophone volume to a few milliliters, shown in Figure 1b. A pair of acoustic resonators made of stainless-steel tubes was added to the bare QTF to improve the QEPAS spectrophone performance. In this work, the most compact QEPAS spectrophone ever, which was 3.7 times smaller than the standard QTF, was employed for trace gas sensing. A picture of a micro QTF and a usually used standard QTF are shown in Figure 2a. The dimensions of two QTFs were obtained by a stereoscopic microscope with a 40× lens.

The geometry of the micro QTF and standard QTF are marked in Figure 2b and listed in Table 1. The prong width *w*, thickness *t*, and length *l_1_* of micro QTF are 56%, 39%, and 27% less than the standard QTF. As a result, the effective prong weight of micro QTF is 80% less than the standard QTF considering that the density of the two QTFs are similar. A prong spacing of 200 μm of the micro QTF is 33% narrower than that of the standard QTF, resulting in higher acoustic pressure in the collection of acoustic waves [37].

## 3. Quartz-Enhanced Photoacoustic Sensor

**Figure 3:**
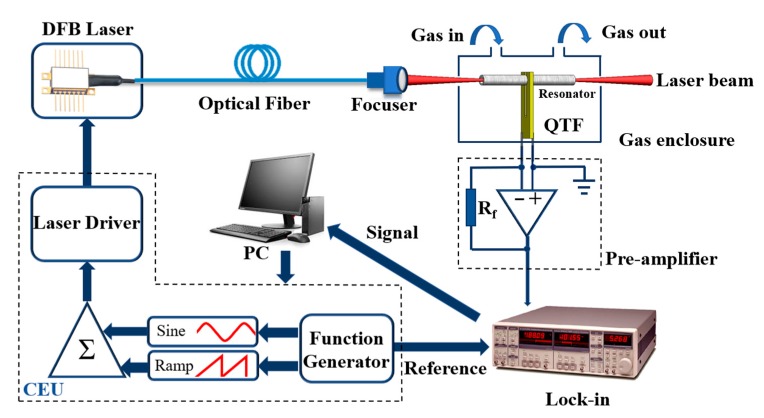
Schematic diagram of the QEPAS system based on a micro QTF. DFB: Distributed feedback; QTF: Quartz tuning fork; R_f_: Feedback resistance; PC: Personal computer; Lock-in: Lock-in amplifier; CEU: Control electronic unit; ∑: adder.

As a proof of concept, a QEPAS system based on a micro QTF for H_2_O detection is depicted in Figure 3. A LabView program (National Instrument) was used to control the system. A 1.39 μm near-infrared fiber coupled distributed feedback (DFB) diode laser (NTT Electronics) is used as an excitation source. The pigtail style focuser (OZ Optics) has an *f* = 1.01 mm GRIN lens in a 2.5 mm out diameter housing with 40 dB return loss. The 9/125 single mode fiber was terminated with an angled FC/APC connector. The beam quality was shown in our previous publication [38]. A custom control electronic unit (CEU) [20] was employed as the laser driver to control the temperature and injection current of the diode laser. A ramp signal with a frequency of 10 mHz and a sine signal with a frequency of *f*_0_*/*2, generated from the waveform function generator module were added to the laser driver, where *f*_0_ is the resonance frequency of the QTF. The use of a 2*f* wavelength modulation technique in QEPAS increases the detection sensitivity. The laser beam was focused through the QTF prong spacing by means of a grin lens. The diameter of the beam waist was ~100 μm at a working distance of 1 cm. A good beam quality guarantees that the laser beam passes through the QTF prong spacing without touching the QTF. The acoustic waves generated by the photoacoustic effect results in the vibration of the QTF. The electrical signal generated by the QTF is amplified by a custom transimpedance preamplifier with a 10 MΩ feedback resistor and sent to a lock-in amplifier (Standford SR830), which demodulates the signal in a 2*f* mode. Finally, the LabView program displays the calculated H_2_O vapor concentrations from the QEPAS 2*f* signal.

## 4. Improvement of Laser Excitation of the Micro Quartz Tuning Fork

**Figure 4:**
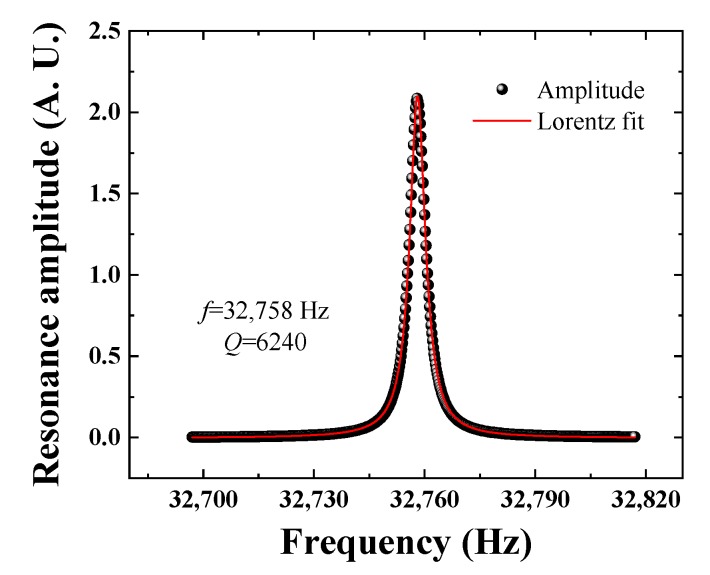
Schematic diagram of the QEPAS system based on a micro QTF.

The electrical parameters of the micro QTF were obtained using the same circuit as reported in our previous publication [20]. A function generator (AFG3102, Tektronix, Beaverton, OR, USA) was used to provide a sine signal to drive the QTF. The frequency of the sine signal was scanned from 32,697 Hz to 32,817 Hz in steps of 0.02 Hz. The peak to peak amplitude of the sine signal was set to 300 mV. A lock-in amplifier (SR830 DSP, SRS, Inc., Hendersonville, TN, USA) was used to demodulate the QTF output signal. The frequency response of the QTFs were recorded by a personal computer (PC). The Q factor here is defined by *f*/Δ*f*, where *f* is QTF resonance frequency and Δ*f* is the full line width at half maximum (FWHM) of the resonance curve. The Lorentz function was used to fit the response curve to obtain the FWHM of 5.25 Hz. Figure 4 shows the resonance curve of the micro QTF. The resonance frequency *f*_0_ and the Q factor obtained from the Lorentz line fitting were 32,758 Hz and 6240, respectively. Due to the thermal stability properties of the quartz, the resonance frequency and Q factor of a bare QTF remains almost constant in the atmospheric conditions [39].

**Figure 5:**
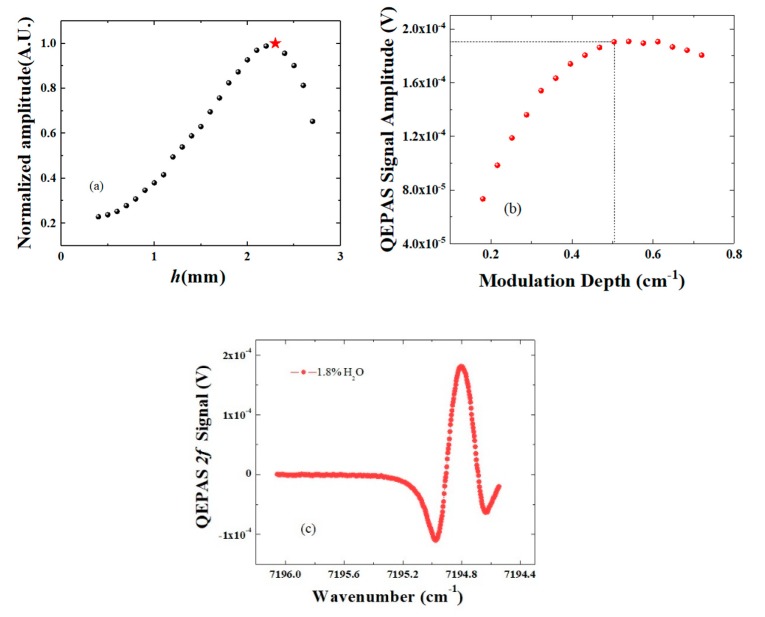
Improvement of laser excitation to the micro quartz tuning fork. (**a**) Normalized QEPAS signal amplitude as a function of laser focus position *h*; (**b**) QEPAS signal amplitude as a function of laser modulation depth; and (**c**) maximum QEPAS 2*f* signal with the optimized laser focus height and modulation depth.

The QTF prongs can be approximated as a cantilever with a fixed end, and the QEPAS signal is related to the moment of force of the acoustic sources. The acoustic waves are cylindrical and there are position effects between the laser beam and the quartz tuning fork. To obtain the maximum QEPAS signal of the micro QTF, the height (*h*) of the laser focus position was changed from the bottom of the QTF (*h* = 0) to the opening of the QTF (*h* = 2700 μm). The laser temperature and injection current were set to 17.5 °C and 50 mA, respectively. The laser power from the fiber focuser was measured to be 5.8 mW by using of a power meter (Ophir Nova II). The normalized signal amplitude as a function of *h* was shown in Figure 5a. The maximum QEPAS signal amplitude was achieved at *h* = 2.3 mm, which is 0.4 mm from the QTF opening. This is different from that of the standard QTF and a custom large QTF.

The modulation depth describes how much the laser wavelength varies around its mean value. According to the theory of wavelength modulation spectroscopy, the laser modulation depth maximizing the QEPAS signal should be optimized [40]. The laser modulation depth was varied from 5 mA to 20 mA, corresponding to the wavenumber from 0.18 cm^−1^ to 0.72 cm^−1^. The QEPAS signal amplitude as a function of laser modulation depth is shown in Figure 5b. It was found that the signal amplitude increased by <1% after 0.504 cm^−1^ and started to decrease when the modulation depth is larger than 0.612 cm^−1^. As a result, the optimum laser modulation depth was ~0.504 cm^−1^, which corresponds to the laser current as 14 mA. With the optimal conditions of *h* = 2.3 mm and a modulation depth of 0.504 cm^−1^, the maximum QEPAS 2*f* signal is showed in Figure 5c. The laser wavelength was tuned from 15 mA to 60 mA, corresponding to the wavenumber from 7196 cm^−1^ to 7194.5 cm^−1^, to cover the H_2_O absorption line falling at 7194.8 cm^−1^ with an intensity of 3.07 × 10^−21^ cm/mol. Since the threshold of the laser was ~2 mA, the laser was partially turned off in the region of 15 mA~16 mA. The 1σ noise level calculated from the absorption line was 1.08 μV, resulting in a signal-to-noise ratio (SNR) of 286. The water vapor concentration was controlled to be 1.8% by a humidifier (PermSelect) [41]. The measurement was carried out at room temperature ~25 centigrade and atmospheric pressure.

## 5. Optimization of Sensor Configuration Based on a Micro Quartz Tuning Fork

**Figure 6:**
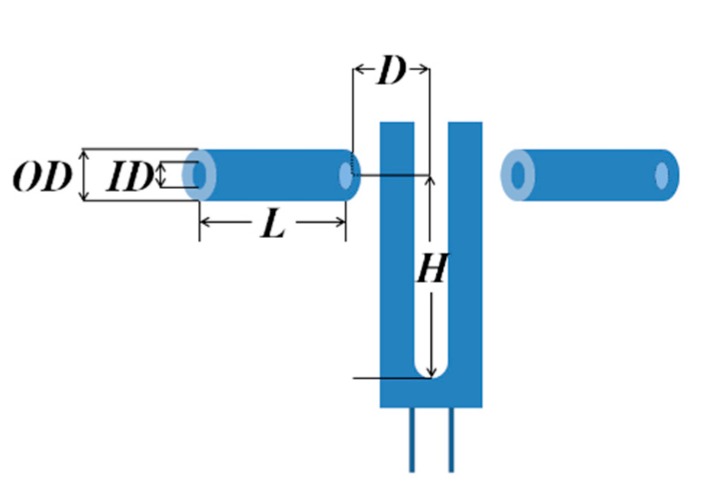
Schematic diagram of micro quartz tuning fork configured with acoustic micro resonators in an on-beam configuration.

In order to improve the performance of a bare micro QTF, acoustic micro resonators (AmR) were configured with the bare QTF to confine the acoustic waves and amplify the resonance. As shown in Figure 6, *OD* is the outer diameter of AmR, *ID* is the inner diameter of AmR, and *L* is the AmR length, respectively. *D* is the gap between the AmR and QTF and *H* is the height of the AmR, respectively.

Five stainless-steel capillaries with different geometries were selected to construct the acoustic micro resonators (AmR). The geometrical parameters of the AmRs are listed in Table 2.

**Figure 7:**
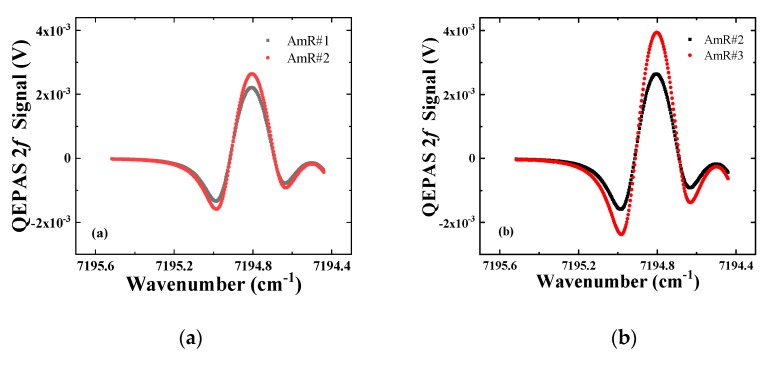

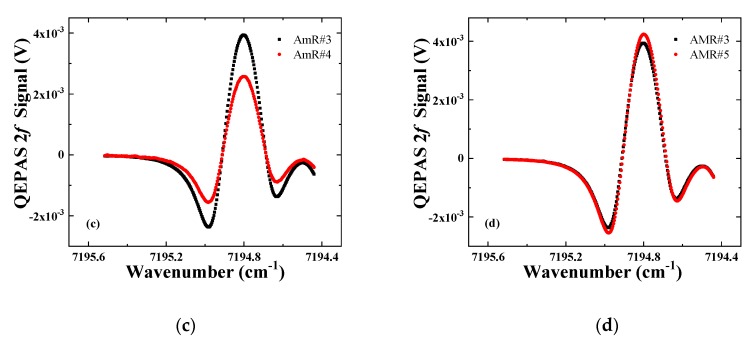
Optimization of the sensor configuration. (**a**) Optimization of the AmR length *L*; (**b**) optimization of the AmR inner diameter *ID*; (**c**) optimization of the gap distance *D;* (**d**) optimization of the assembly height *H*.

According to the theory of a one-dimensional acoustic resonator, the AmR length has a significant impact on the resonance of acoustic waves. For an ideal one-dimensional acoustic resonator, the resonance frequencies can be estimated by the standing wave equations with the approximated end correction [42]. However, for a QEPAS on-beam configuration, a micro QTF with a thickness of 200 μm was inserted in the middle of the ideal one-dimensional acoustic resonator. The insertion of the QTF distorted the acoustic pressure distribution and formed a coupled QEPAS system. Therefore, it is difficult to calculated resonance frequency and Q factors of the coupled QEPAS system precisely. However, experiential optimization of geometry parameters of the standard QEPAS system can be obtained from our previous publication [27]. Here, the optimum length of the AmR was researched by experiment. AmR#1 and AmR#2 with the length of 4 mm and 4.4 mm were selected for comparison. The obtained signal of AmR #2 is 19% greater than AmR #1, as shown in Figure 7a. Since the pair of AmRs was separated by the QTF, the optimum AmR length was slightly longer than the half of the acoustic wavelength [27,36].

The inner diameter (*ID*) of the resonator determines the resonant acoustic modes. With an AmR length of 4.4 mm, the inner diameter of AmRs was optimized. AmR #2 and AmR #3 with an *ID* of 0.7 mm and 0.6 mm were selected. Figure 7b depicts the QEPAS signals obtained by a micro QTF configured with AmR #2 and AmR #3, respectively. The signal obtained by a micro QTF configured with an AmR #3 is 49% higher.

In the case of QEPAS based on a standard QTF, the effect of the gap between the tubes and QTF was researched theoretically [43]. The theoretical results showed that the photoacoustic signal decreases with an increasing gap, since a larger gap causes more acoustic energy leakage from the gap and reduces the acoustic coupling between the AmRs. For traditional standard QTF, the optimized gap *D* was ~20 μm. However, for the micro QTF, the viscous damping becomes significant with a *D* of 20 μm, resulting in a Q factor <1000 due to the small effective weight of the QTF. With *D* increasing to 160 μm, the QEPAS signal amplitude decreased by 35%, as shown in Figure 7c. Here, *D* = 80 μm was a tradeoff.

Finally, the position effects of the AmR was taken into consideration. The research process is similar to that for the standard QTF and the custom QTF as reported in our previous publications [44,45]. With the *ID* = 0.6 mm, *L* = 4.4 mm, and *D* = 0.08, the height *H* of AmR was scanned along the QTF prong. The QEPAS signal obtained by *H* = 2.5 mm and 2.3 mm is plotted in Figure 7d. The results revealed that the optimum *H* was ~2.3 mm, which coincides with the optimum height of the laser focus spot. The enhancement induced by *H* was moderate, since there is a flat section in the AmR position effects.

## 6. Evaluation of the QEPAS Sensor Based on Micro QTF

**Figure 8:**
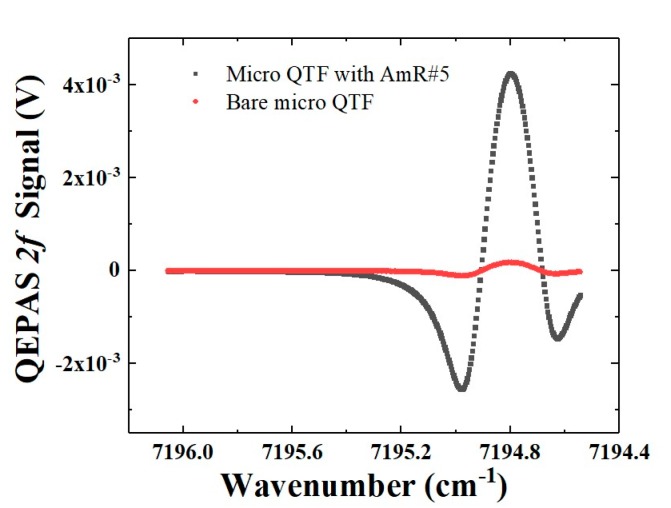
QEPAS 2*f* signal based on H_2_O measurements when using a bare micro QTF and a micro QTF with an AmR in on-beam configuration.

The QEPAS sensor performance based on a bare micro QTF and an on-beam configuration were compared. Figure 8 depicts the 2*f* signals of the sensors based on a bare micro QTF and the on-beam configuration. The laser temperature was set to 17.5 °C. The injection current was scanned from 7196 cm^−1^ to 7194.5 cm^−1^ to target the H_2_O absorption line. In the experiment, the H_2_O concentration was controlled at 1.8% using the same method as reported in our previous publication [41]. The AmR #5 with an optimum length of 4.4 mm, an *OD* of 1 mm, and an *ID* of 0.7 mm was selected. The gap *D* between the AmR and the QTF was set to 0.08 mm. The AmR was positioned at *H* = 2.3 mm from the QTF bottom.

The performance of sensors based on a bare micro QTF and the on-beam configuration are analyzed in Table 3. Due to the acoustic coupling effects between the QTF and the AmR, the Q factor of the QEPAS sensor decreased from 6240 to 4166. For sensors based on a bare micro QTF and the on-beam configuration, the signal amplitudes were 4238 μV and 180 μV, respectively. The 1σ noises standard deviations were calculated with the laser wavelength tuned far away from the targeted H_2_O line. The calculated 1σ noise for QEPAS sensor based on an on-beam configuration was slightly higher than that of the bare micro QTF. This can be attributed to the additional noise caused by the scattered light illumination on the AmR. However, the SNR of 2*f* signal was enhanced by ~10 times by the on-beam configuration. The normalized noise equivalent absorption coefficient (NNEA) of the QEPAS sensor can be calculated by normalizing the minimum absorption coefficient αmin to the laser power (*P_L_*) and the detection bandwidth (Δ*f_L_*) according to the relation:

(1) $$NNEA=\frac{\alpha_{min}P_{L}}{\sqrt{\Delta f_{L}}}$$

With a 1 s integration time and 12 dB/oct filter slope, an NNEA of 1.97 × 10^−8^ W·cm^−1^·Hz^−1/2^ was achieved by the QEPAS sensor based on a micro QTF with an on-beam configuration. This achieved NNEA was one order lower than that of the QEPAS sensor based on a bare micro QTF. The NNEA is comparable with the conventional QEPAS sensor based on a standard QTF with an on-beam configuration. However, the QEPAS sensor based on micro QTF was much more compact.

## 7. Discussion

A small gas sampling volume is always desired in the measurement such as microorganism respiration [47], human breath [48], and atmospheric air [49]. According to the geometry of the QTF, the calculated gas sampling volume of the micro QTF is ~0.1 mm^3^, which is several orders of magnitude smaller than conventional photoacoustic spectroscopy (PAS) sensor [50,51] and tunable diode laser absorption spectroscopy (TDLAS) sensor [52]. For a typical QEPAS system, the gas flow rate was limited to 200 sccm (standard cubic centimeter per minute) in order to avoid gas flow noise [27]. Even such a gas flow rate results a fast system flush time of the QEPAS spectrophone based on micro QTF was ~30 μs. After checking the laser diodes in stock, this experiment was carried out by detecting the water vapor as proof of concept. Further demonstration can be made by analysis the NO_x_ and CO_x_, which will benefit breath analysis for seriously ill patients and infants whose breath is relatively weak.

## 8. Conclusions

In this paper, an ultra-compact QEPAS sensor based on a micro QTF 3.7 times smaller than a standard QTF was reported. As a proof of concept, the performance of QEPAS sensor based on a micro QTF was evaluated by detecting water vapor. A detailed analysis of the laser focus position effects and laser modulation depth were performed to improve the excitation efficiency. A pair of acoustic micro resonators (AmRs) was assembled with the micro QTF in an on-beam configuration in order to enhance the photoacoustic signal. The resonator length and resonator diameters were optimized to obtain strong resonant acoustic modes. The AmRs distance and height with respect to the micro QTF was also investigated in order to obtain the optimum QEPAS signal. As a result, the SNR increased by a factor of ~10 when using the AmR. A normalized noise equivalent absorption coefficient (NNEA) of 1.97 × 10^−8^ W·cm^−1^·Hz^−1/2^ was achieved when detecting H_2_O concentrations of less than 1 atm. For the detection of different gases, the laser wavelengths should be changed. 

The use of a micro QTF was helpful in the signal improvement, since the photoacoustic signal is inversely proportional to the gas volume holding by the QTF prong spacing [20]. In addition, the acoustic waves decreased significantly in the vicinity of laser beam. The prong spacing of the used micro QTF is 33% smaller than the usually used standard QTF, resulting in the micro QTF being more efficient in the collection of photoacoustic waves. With respect to the analysis time, in a QEPAS system, the time needed to analyze the sample is mainly determined by the volume of the gas chamber; a more compact gas chamber assembled with the micro QTF will reduce the time needed to analyze a gas sample significantly. The advantages of QEPAS sensor is that the intrinsic property of QTF such as resonance frequency is not sensitive to the environmental temperature and humidity [4,39]. The instrument based on the micro QTF with a small gas sampling volume and fast response time could benefit breath measurements for seriously ill patients and infants whose breath is relatively weak. Although we selected a weak absorption line in the laser wavelength range for the detection of H_2_O as proof of concept, the detection limit of the sensor is acceptable when compared to the recently developed optical fiber sensor [53] or graphene sensor [54]. The Knudsen effect [55] and a feedback method can be further considered to improve the performance of the sensor based on micro QTF [56]. The micro QTF can also be used as a detector for stand-off detection of explosives as well as a laser vibrometer [57]. Photothermal analysis can also be achieved by using of the micro QTF [58,59,60].

## Figures and Tables

**Table 1:** Geometric parameters of micro QTFs and standard QTFs. The *w*, *g*, *t*, *l*_1_, and *l*_2_ represent the prong width, prong spacing, prong thickness, prong length, and tuning fork length, respectively.

	*w* (μm)	*g* (μm)	*t* (μm)	*l*_1_ (μm)	*l*_2_ (μm)
Micro QTF	260	200	200	2700	3500
Standard QTF	600	300	330	3700	6200

**Table 2:** The geometrical parameters of AmRs.

AmR	*OD* (mm)	*ID* (mm)	*L* (mm)	*D* (mm)	*H* (mm)
AmR #1	1	0.7	4	0.08	2.5
AmR #2	1	0.7	4.4	0.08	2.5
AmR #3	1	0.6	4.4	0.08	2.5
AmR #4	1	0.6	4.4	0.16	2.5
AmR #5	1	0.6	4.4	0.08	2.3

**Table 3:** The QEPAS sensor performance based on bare micro QTF and an on-beam configuration. AmR #5 was selected as the resonator. SNR: Signal-to-noise ratio; NNEA: Normalized noise equivalent absorption coefficient.

	Q Factor	Signal (μV)	1σ Noise (μV)	SNR	NNEA (W·cm^−1^·Hz^−1/2^)
Bare micro QTF	6240	180	0.63	286	1.74 × 10^−7^
Micro QTF with an on-beam configuration	4166	4238	1.66	2553	1.97 × 10^−8^
Standard QTF with an on-beam configuration [46]					1.68 × 10^−8^
